# On Spermatorrhœa: Its Causes, Symptomatology, Pathology, Prognosis, Diagnosis, and Treatment

**Published:** 1866-09

**Authors:** 


ON SPERMATORRHŒA: ITS CAUSES, SYMPTOMATOLOGY, PATHOLOGY, PROGNOSIS, DIAGNOSIS, AND TREATMENT. By ROBERTS BARTHOLOW, A.M., M.D., Prof. of Physic and Med. Chemistry, in Med. College of Ohio; Lecturer on Clinical Medicine, and Physician to St. John's Hospital, Cincinnati, etc., etc. New York: WM. WOOD & Co., 61 Walker Street. 1866.

This is a very neatly printed monograph of 112 pages. The style of the author is clear, concise, and pleasing; and his practical views judicious. In regard to the special pathology of spermatorrhœa, he says:—"The pathological conditions may be comprehended in three groups—genital; cerebral; spinal. In the first, or genital form or phase, which is the most common, there are excessive sensibility of the sexual apparatus, and greatly increased reflex excitability of the cord. In the cerebral form, there are associated with the preceding condition, certain disorders of the mind—melancholia, delusional insanity, and mania. In the spinal form, the functional derangement of the cord is either excessive and pronounced, or has resulted in organic lesion." With such diversity in the pathology of different cases, there must follow equal variations in the treatment. Hence, the seeker after specifics will find no comfort in reading this work. The rational practitioner, however, will find it worthy of a place on his table.

